# Gender Disparity of First Authors in Review Article Publications Related to Schizophrenia

**DOI:** 10.7759/cureus.47757

**Published:** 2023-10-26

**Authors:** Vetrivel Vijayakumar, Hannah Fathima Babu, Aakriti Karki, Rahul Tyagi, Myla Macapia, Kathryn M Zapata, Srikanth Dogiparthi

**Affiliations:** 1 Internal Medicine, American University of Barbados School of Medicine, Bridgetown, BRB; 2 Internal Medicine, K.S. Hegde Medical Academy, Nitte University, Mangalore, IND; 3 Internal Medicine, Jalalabad Ragib-Rabeya Medical College, Sylhet, BGD; 4 Family Medicine, Leeds Confederation of General Practitioners, Leeds, GBR; 5 Family Medicine, Royal College of General Practitioners, London, GBR; 6 Internal Medicine, NYC Traumatic Brain injury Center, New York, USA; 7 Internal Medicine, Ross University School of Medicine, Bridgetown, BRB; 8 Department of Medicine, Pontiac General Hospital, Michigan, USA

**Keywords:** gender equity, academic publishing, schizophrenia, review articles, first authors, gender disparity

## Abstract

Schizophrenia is a severe psychotic condition that can be diagnosed when certain symptoms, such as disorganized speech, disorganized thoughts, or negative feelings, are present for at least six months in a person's life. Gender equity and representation in academic writing are significant issues that have received more attention recently. Understanding the gender discrepancies in authorship can help researchers studying schizophrenia overcome obstacles and potential biases. The purpose of this study was to determine the degree of gender discrepancy among initial authors of articles that focused on schizophrenia and to identify potential causes of such inequalities. A bibliometric analysis of articles related to schizophrenia published from 2019 to 2022 was conducted. The authors' genders were determined through available public records and professional affiliations. The analysis included assessing the proportion of male and female first authors and examining trends over time. A total of 982 articles were included in the analysis. The results revealed a significant gender disparity in first authorship, with a higher representation of male first authors (546, 55.6%) compared to female first authors (436, 44.4%). There is a significant increase in the percentage of female authors from 2019 to 2022 (i.e., from 25% to 48.5%). The number of female and male authors is predicted to remain at a constant from 2023 to 2027, with male authors at 175 and female authors at slightly above 150. The findings of this study underscore the need for continued efforts to address gender imbalances in academic publishing and promote gender equity in the field of schizophrenia research. Recognizing and rectifying these disparities can contribute to a more inclusive and diverse scientific community.

## Introduction and background

Schizophrenia is a severe psychotic condition that can be diagnosed when certain symptoms, such as disorganized speech, disorganized thoughts, or negative feelings, are present for at least six months in a person's life. Hallucinations and delusions are two of the hallmarks of schizophrenia. Approximately one in 10,000 persons are diagnosed with this psychiatric disorder each year [1]. This indicates that there is a substantial quantity of scholarly interest and publications devoted to this disease. In spite of the enormous amount of interest that has been shown in schizophrenia by the academic community, it is common knowledge that there are significant gender gaps in the composition of research articles of any kind, both as authors and as editors [2]. Not only that, when compared with male physician writers, the number of research publications authored by female physicians in the first and final author positions is much lower. Women also co-authored articles with other women, although this occurs significantly less frequently [3].

Even though there has been a significant rise in the number of women working in medicine in general and in psychiatry in particular over the course of the past 40 years, there are still insufficient opportunities for women to progress their careers in academic medicine [4]. Psychiatric journals continue to boost the representation of female writers, leading to near parity among initial authors. However, slower rates of advancement to senior authorship and a persistent underrepresentation of female senior authors imply that reaching gender equity in academic leadership will continue to be difficult. Women would reach parity in senior authorship in 20 to 25 years at the current pace of change for the last authors (0.64% rise each year) [5]. 

The purpose of this analysis is to quantify and characterize the scope of gender variations in initial authorship in research pertaining to schizophrenia, one of the most thoroughly investigated neuropsychiatric disorders in the medical literature.

The aim of this research was to analyze and assess the trends in the gender of first authors in schizophrenia publications from 2020 to 2022. The objectives of this study were to determine the ratio between male and female first authors, identify the leading countries and journals with significant gender ratios, and make predictions about the potential trends for the next five years.

## Review

A bibliometric analysis of articles related to schizophrenia published over the last three years was conducted. A search was done on PubMed with the keyword "schizophrenia," which resulted in 989 papers from January 2020 to December 2022. These 989 papers were entered in a Google sheet with details, such as authors, citation, first author, country of the first author, journal/book, publication year, creation date, PubMed Central identification (PMCID), NIH Manuscript Submission (NIHMS) ID, and digital object identifier (DOI) (if available). These papers were divided equally among the authors and each author then entered the gender of the first author. The gender of the first author was found from a general online search and sometimes specifically from a search on Namsor.

Statistical analysis was then conducted on this data using the IBM SPSS Statistics for Windows, version 21 (releases 2012, IBM Corp., Armonk, New York, United States). The total number of male and female authors by year was calculated. Forecasting for publications by male and female authors was done using single exponential smoothing from 2022 to 2027 using data from 2020 to 2022. The top 10 journals with at least five publications and a favorable gender ratio were shortlisted. Moreover, the top 10 countries with at least 10 publications and a favorable gender ratio were calculated.

A total of 988 scholarly articles pertaining to schizophrenia were analyzed, out of which 984 articles provided complete information regarding the full names of the authors. This information was critical to the study as it facilitated the identification and categorization of the authors' genders. Six authors for whom gender could not be assigned due to ambiguity of the names and insufficient publicly available information were excluded from subsequent analysis. The final dataset, therefore, included 982 authors, allowing for a comprehensive comparative analysis of gender disparity among authors in the field of schizophrenia research.

**Figure 1:**
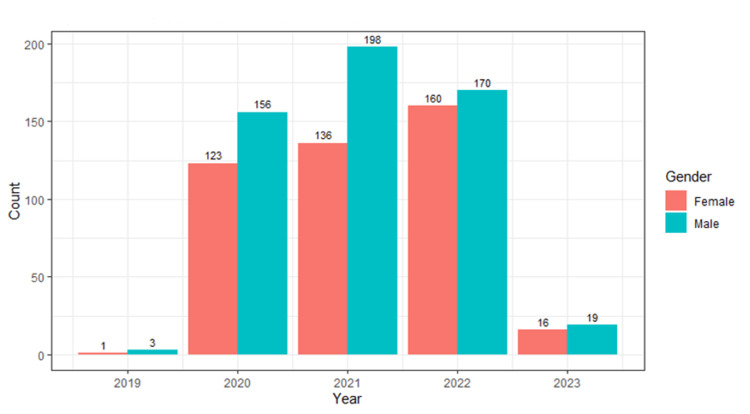
Total number of male and female authors each year from 2020 to 2023. Some articles were accepted in 2019; pre-prints or online versions were available for some in 2019 or 2020 and finally published in 2020 or later. These articles appeared in our search on PubMed and hence included in 2019.

Figure 1 shows the total number of male and female authors each year from 2020 to 2023. In all years, the female first authors were less as compared to male first authors.

A total of 982 first authors were identified, among whom 436 (44.4%) were female and 546 (55.6%) were male. The percentage of male authors ranged from 51.5% to 59.3% across the years, while the percentage of female authors ranged from 40.7% to 48.5%. There is a significant increase in the percentage of female authors from 2020 to 2022 from 25% to 48.5%.

**Figure 2:**
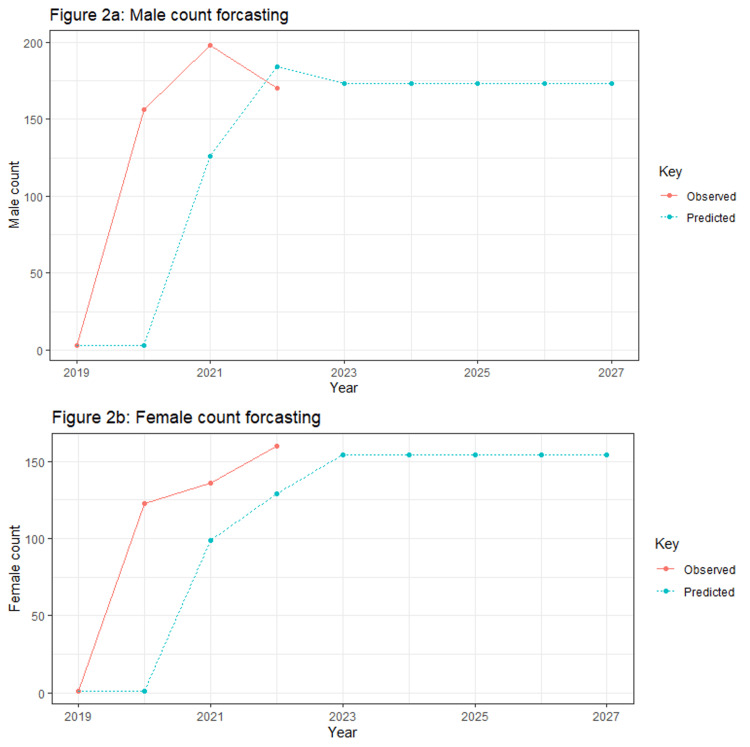
Observed publication trends among the male count (a) and female count (b) first authors from 2020 to 2022 and the prediction of trends for the next five years.

Figure 2a and 2b display the observed publication trends among the male count (a) and female count (b) first authors from 2020 to 2022 and the prediction of trends for the next five years. The number of female and male authors is predicted to remain at a constant from 2023 to 2027, with male authors at 175 and female authors at slightly above 150.

**Figure 3:**
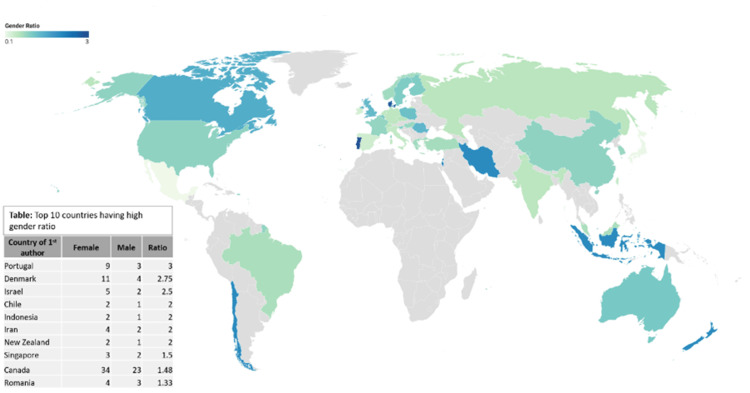
Gender trends in publications (2020-2023) based on country. Image created by the authors using Datawrapper software

Figure 3 shows the top 10 countries having a high gender ratio of first authors. The gender ratio of first authors was the highest for Portugal (3), followed by Denmark (2.75) and Israel (2.5).

Table 1 shows the top countries having a high gender ratio of first authors. Each country must have a threshold of minimum of 10 publications. The analysis of first authors from various countries reveals variations in gender representation within the research community. The gender ratios, representing the number of female first authors compared to male first authors, range from 3 to 0.71 across the studied countries.

**Table 1:** Top countries having a high gender ratio (at least 10 publications)

Country of first authors	Female	Male	Ratio
Portugal	9	3	3
Denmark	11	4	2.75
Canada	34	23	1.48
United Kingdom	33	26	1.27
Poland	20	17	1.18
Australia	21	20	1.05
China	34	38	0.89
USA	98	111	0.88
France	17	20	0.85
Turkey	5	7	0.71

Countries, such as Portugal, Denmark, and Canada, demonstrate gender ratios above 1, indicating a relatively higher representation of female first authors. Portugal stands out with the highest gender ratio of 3, followed by Denmark with 2.75 and Canada with 1.48. The United Kingdom, Poland, Australia, and China showcase gender ratios slightly above 1, indicating a slightly higher representation of female first authors. These countries demonstrate a range of gender ratios from 1.27 (United Kingdom) to 1.05 (Australia). By contrast, the USA, France, and Turkey exhibit gender ratios below 1, indicating a higher representation of male first authors. The USA and France have gender ratios of 0.88 and 0.85, respectively, while Turkey has a gender ratio of 0.71. Fischer's exact test was performed to determine the statistical significance between the gender and country variables, and p-value was found to be 0.0004998, which signifies that there is a significant association between gender and country.

Table 2 shows the top journals having favorable gender ratio of first authors, a threshold of at least five publications. Favorable gender ratio signifies near equal male and female first authors. *Current Opinion in Psychiatry* has the highest gender ratio of 1.43, while *Neuroscience* and *Biobehavioral Review* has the lowest, i.e., 0.77.

**Table 2:** Top journals having favorable gender ratios (at least five publications) Curr Opin Psychiatry: Current Opinion in Psychiatry; Psychol Med: Psychological Medicine; Front Psychiatry: Frontiers in Psychiatry; Schizophr Res: Schizophrenia Research; Biol Psychiatry: Biological psychiatry; Expert Rev Neurother: Expert Review of Neurotherapeutics; Front Pharmacol: Frontiers in Pharmacology; Int J Mol Sci: International Journal of Molecular Sciences; Schizophr Res Cogn: Schizophrenia Research: Cognition; Neurosci Biobehav Rev: Neuroscience & Biobehavioral Reviews

Journal/book	Female	Male	Ratio
Curr Opin Psychiatry	10	7	1.43
Psychol Med	5	4	1.25
Front Psychiatry	30	26	1.15
Schizophr Res	31	29	1.07
Biol Psychiatry	7	7	1
Expert Rev Neurother	3	3	1
Front Pharmacol	4	4	1
Int J Mol Sci	14	17	0.82
Schizophr Res Cogn	4	5	0.8
Neurosci Biobehav Rev	17	22	0.77

Table 3 shows top journals having high gender ratios. For consideration, each journal should have had a threshold of minimum five publications. *Biological Psychiatry* and *Frontiers in Pharmacology* demonstrate an encouraging equal gender representation with gender ratios of 1. In these journals, the proportion of female first authors is equivalent to that of male first authors.

**Table 3:** Top journal having high gender ratios (at least five publications) Brain Sci: Brain Sciences; J Psychiatr Res: Journal of Psychiatric Research; Front Neurosci: Frontiers in Neuroscience; Behav Sci (basel): Behavioral Sciences; Curr Opin Psychiatry: Current Opinion in Psychiatry; Psychol Med: Psychological Medicine; Front Psychiatry: Frontiers in Psychiatry; Schizophr Res: Schizophrenia Research; Biol Psychiatry: Biological Psychiatry; Front Pharmacol: Frontiers in Pharmacology

Journal/book	Female	Male	Ratio
Brain Sci	4	1	4
J Psychiatr Res	9	3	3
Front Neurosci	9	5	1.8
Behav Sci (basel)	3	2	1.5
Curr Opin Psychiatry	10	7	1.43
Psychol Med	5	4	1.25
Front Psychiatry	30	26	1.15
Schizophr Res	31	29	1.07
Biol Psychiatry	7	7	1
Front Pharmacol	4	4	1

Among the top journals with favorable gender ratios, *Current Opinion in Psychiatry* stands out with a ratio of 1.43, indicating a slightly higher representation of female authors compared to male authors. This trend is also evident in *Psychological Medicine* (ratio: 1.25), *Frontiers in Psychiatry* (ratio: 1.15), and *Schizophrenia Research* (ratio: 1.07). Meanwhile, a few top journals exhibit a notably higher representation of female authors compared to male authors. *Brain Sciences*, with the highest gender ratio of 4, and *Journal of Psychiatric Research* (ratio: 3) lead the way in providing a platform for female researchers to contribute their valuable insights.

The main goal of this study is to describe changes in gender disparity of first authors in publications related to schizophrenia from 2020 to 2022. Even though gender disparity is seen in various kinds of fields, such as education, medicine, healthcare and sports, our study shows its implication in research and publications related to schizophrenia. This study revealed that, in the year 2020, 44% (123) of first authors were females, followed by 40% (136) in 2021 and 48% (160) in 2022. This shows that gender differences are present in achieving first authorship between men and women. In each year, the number of males is comparatively more than the number of females as first authors. Although the proportion of women as first authors increased from 2020 to 2022, they stayed behind as minority authors every year. This finding is homogeneous to the study of gender disparity in publications within surgical subspecialities by Sela et al. [6].

The authors have also identified that the overall male and female first authors in publishing schizophrenia-related articles in the year 2020 are relatively lower compared to those in the next two years. This may be due to the COVID-19 pandemic where most of the researchers shifted their focus on COVID-based research publications. This statement is justified by a study that shows how the COVID-19 pandemic had a severe impact on non-COVID-related publications [7]. 

As far as we know, there are no existing data on predictions of future gender disparity related to schizophrenia publications until now. The observed future trends in our study for the next five years of male and female as first authors predicted by exponential forecasting showed that the male count plateaued nearby 175 (53%) and the female count plateaued nearby 155 (47%) starting from the year 2023. It clearly shows that the gender disparity will be existing at the average difference of approximately 20 (6%) between male and female counts as first authors for the next five years in schizophrenia relevant publications. Further investigations in the future are needed to examine and support the obtained results.

Attaining first authorship in publications explains the amount of participation and leading skills of the author. The studies performed by Hafeez et al. [8] and Madden et al. [2] shows that there are fewer women as first authors, editorial head or advisory board members of psychiatry journals and other medical journals worldwide, which shows the lack of women in leadership positions [8,9]. There are several factors contributing to these disparities, such as family obligations, workplace environment, sex discrimination, and lack of female mentors. This must be addressed appropriately to lead and motivate women in their professional growth.

Gender disparity based on country

It is noteworthy that the first authors' genders varied inevitably based on geographic locations. As per our observation, the first author's gender is significantly associated with their country of origin (p-value = 0.0005). For example, most of the countries in Europe and North America have the highest female first authors with a high gender ratio having at least 10 publications. These results are underpinned by the studies conducted by He and Gong [10], where they analyzed the female authorship trends in the field of colorectal surgery. Similar findings were noted in the study inspected by Phurtag et al. [11], which indicated the highest contribution of female authors from Europe and North America in spine-related journals. On a country level, Portugal has the highest female first authors with a gender ratio of three, and Australia has a near-favorable gender ratio where you can find almost equivalent male and female first authors. It is because of the fact that women in developed countries are more educated, are provided equal opportunities, and have fewer social stereotypes. On the contrary, our study revealed that some developed countries, such as the USA and France, still dominate with slightly higher first male authors with a low gender ratio. This small gender gap may eventually close in the upcoming years if more priority is given toward gender equality. We also noticed some developing countries in Asia, such as Iran and Indonesia, having the highest female first authors too, which shows their efforts in achieving gender equity. In fact, a study based on women’s authorship in an Iranian medical journal from 1999 to 2019 proved that the role of women as first authors increased from 26.7% to 54.9%, which supports our results observed in developing countries [12].

Gender disparity based on journals

The authors also analyzed the gender of first authors in various journals worldwide related to schizophrenia. We identified several top journals showing a favorable gender ratio (around 1), which is a comparatively equal gender count. We found that journals with a good impact factor, such as *Biological Psychiatry*, *Expert Review of Neurotherapeutics,* and *Frontiers in Pharmacology,* show exactly equal gender counts as first authors. This finding is promising against gender inequality, and the closing of gender gap is evidently seen in these journals, and it was as expected in the study of Abraham et al. that examined the observed closing gender gaps for first authors over the last decade in high-ranking US nephrology journals [13]. Dissimilarly, we also noticed that not-high- but good-impact-factor journals, such as *Brain Sciences*, *Journal of Psychiatric Research,* and *Frontiers in Neuroscience*, have the highest number of first female authors with at least five publications related to schizophrenia. Of note, there are some high-impact-factor specialty-specific journals where a decreased rate of first female authors was observed [14], and there are some high-impact-factor specialty-specific journals where an increased rate first female authors was noticed [15]. This shows that there are varying results of gender disparity detected in different studies based on the type of journals, specialties, and research topics.

Limitations

There are few limitations present in this study. Primarily, the data collection from the website called “Namsor.app” where we analyzed the gender of first authors based on information, such as author first name, author last name, and country of origin, could lead to a minor possibility of misclassification bias due to the name-based data collection process. Even though the contribution of this bias is relatively small, the most accurate data can be collected by mentioning the gender of the first author in every publication. Next, the data were collected only for the past three years excluding 2023 and hence may not represent the trends before the studied years, and the use of a chi-square analysis with larger sample size may result in more accurate predictions. Finally, this specific study only represents the gender disparity based on schizophrenia-related articles, so the results may vary if we included articles related to other psychiatric disorders.

## Conclusions

This study reveals a persistent gender disparity in first authorship in schizophrenia research published in academic journals from 2020 to 2022, with male authors predominating. Geographical variations in gender representation and discrepancies among academic journals were also observed. Despite a slight increase in female first authorship, women consistently remain a minority. Predictive models suggest that this gender gap may persist over the next five years, albeit at a smaller difference. These findings highlight the need for continued efforts to address gender imbalances in academic publishing and promote gender equity in schizophrenia research. Continued monitoring of gender trends, implementation of equitable policies, and creation of supportive environments that address gender-specific challenges are vital for fostering a balanced and inclusive research community.

## References

[1] Edinoff A, Wu N, deBoisblanc C (2020). Lumateperone for the treatment of schizophrenia. Psychopharmacol Bull.

[2] Madden C, O'Malley R, O'Connor P, O'Dowd E, Byrne D, Lydon S (2021). Gender in authorship and editorship in medical education journals: a bibliometric review. Med Educ.

[3] Mamtani M, Shofer F, Mudan A, Khatri U, Walker R, Perrone J, Aysola J (2020). Quantifying gender disparity in physician authorship among commentary articles in three high-impact medical journals: an observational study. BMJ Open.

[4] Hart KL, Frangou S, Perlis RH (2019). Gender trends in authorship in psychiatry journals from 2008 to 2018. Biol Psychiatry.

[5] Eigenberg HM, Whalley E (2015). Gender and publication patterns: female authorship is increasing, but is there gender parity?. Women Crim Justice.

[6] Sela N, Anderson BL, Granatowicz AT, Jezewski E, Hoffman AL (2021). Gender differences in authorship among hepato-pancreatico-biliary surgeons. HPB (Oxford).

[7] Lapostolle F, Petrovic T, Goix L, Adnet F (2021). Impact of COVID-19 pandemic on non-COVID-19 publications. Resuscitation.

[8] Hafeez DM, Waqas A, Majeed S (2019). Gender distribution in psychiatry journals' editorial boards worldwide. Compr Psychiatry.

[9] Bendels MH, Müller R, Brueggmann D, Groneberg DA (2018). Gender disparities in high-quality research revealed by Nature Index journals. PLoS One.

[10] He S, Gong J (2023). Female authorship trends in the field of colorectal surgery: a retrospective bibliometric study. Heliyon.

[11] Phurtag RD, Häckel S, Benneker LM, Liu KB, Albers CE, Ahmad SS, Deml MC (2022). Gender authorship trends in spine research publications - Research across different countries from 1976 to 2020. Brain Spine.

[12] Shamsi A, Behboudi E, Barghi M, Heidari H (2023). Trends of women's authorship in an Iranian medical journal from 1999 to 2019. Health Care Women Int.

[13] Abraham RR, Adisa O, Owen ME, Iqbal F, Sulaiman K (2023). Evaluation of gender trends in first authorship in nephrology publications in four major US journals in the last decade. J Nephrol.

[14] Karaman İGY, Gündüz T, Kaçar CY (2022). Is women's place beyond the glass ceiling? The gender gap in academic psychiatry publications in Turkey. Noro Psikiyatr Ars.

[15] Hiller KP, Boulos A, Tran MM, Cruz AI Jr (2020). What are the rates and trends of women authors in three high-impact orthopaedic journals from 2006-2017?. Clin Orthop Relat Res.

